# Improvement in detection of minor alleles in next generation sequencing by base quality recalibration

**DOI:** 10.1186/s12864-016-2463-2

**Published:** 2016-02-27

**Authors:** Shengyu Ni, Mark Stoneking

**Affiliations:** Department of Evolutionary Genetics, Max Planck Institute for Evolutionary Anthropology, Leipzig, D04103 Germany

**Keywords:** Base quality recalibration, Minor allele detection, Next generation sequencing

## Abstract

**Background:**

Minor allele detection in very high coverage sequence data (>1000X) has many applications such as detecting mtDNA heteroplasmy, somatic mutations in cancer or tumors, SNP calling in pool sequencing, etc., where reads with low frequency are not necessarily sequence error but may instead convey biological information. However, the suitability of common base quality recalibration tools for such applications has not been investigated in detail.

**Results:**

We show that the widely used tool GATK BaseRecalibration has several limitations in minor allele detection. First, GATK IndelRealignment fails to work if the sequence coverage is above a certain level since it then becomes computationally infeasible. Second, the accuracy of the base quality largely depends on the database of known SNPs as the control, which limits the ability of de novo minor allele detection. Third, GATK reduces the base quality of sequence errors at the cost of reducing scores for true minor alleles. To overcome these limitations, we present a novel approach called SEGREG, which applies segmented regression to control sequences (e.g. phiX174 DNA) spiked into a sequencing run. Based on simulations SEGREG improves both the accuracy of base quality scores and the detection of minor alleles. We further investigate sequence error and recalibration parameters by applying a Logarithm Likelihood Ratio (LLR) approach to SEGREG recalibrated base quality scores for phiX174 DNA sequenced to very high coverage, and for mtDNA genome sequences previously analyzed for heteroplasmic variants.

**Conclusions:**

Our results suggest that SEGREG improves base recalibration without suffering the limitations discussed above, and the LLR approach benefits from SEGREG in identifying more true minor alleles, while avoiding false positives from sequencing error.

**Electronic supplementary material:**

The online version of this article (doi:10.1186/s12864-016-2463-2) contains supplementary material, which is available to authorized users.

## Background

Next generation sequencing is nowadays routinely applied in almost every field of biomedical research [1]. To cope with the resulting high-throughput but error prone reads, the base quality [2], which corresponds to the position-specific error probability, is widely used and accepted. Unfortunately, the raw base quality from the Illumina default basecaller (Bustard) is inaccurate [3]; thus a number of basecallers aimed at achieving better performance have been developed. They either apply a model-based strategy (e.g., AYB [4], naiveBayescall [5]) or use supervised learning approaches with an additional training set such as phiX174 reads spiked into the run (e.g., Ibis [6], Freeibis [7]). These approaches in general give a more accurate base quality as well as introduce fewer sequencing errors, but they require access to the fluorescence intensity data, which is often discarded since the storage facilities for such data is beyond the capacity of most laboratories. Alternative base quality recalibration tools such as GATK [8] and ReQON [9] attempt to find the error pattern from the raw base quality, and reassign each base a recalibrated base quality to reflect the real error probability. However, these tools mostly are designed for typical coverage data and are untested for minor allele detection in very high sequence coverage (>1000X).

There are many applications involving high sequence coverage data where minor alleles are of biological interest, e.g. heteroplasmy in mitochondrial DNA [10], heterozygous alleles in polyploidy organisms or in pool sequencing [11], and somatic mutations with low minor allele frequency (MAF) in early development in cancers that change over time [12]. The detection of mtDNA heteroplasmy has developed extensively in recent years, from simply setting a cutoff on read counts that satisfy some base quality requirement (e.g. phred score >20) [10, 13–15] to more sophisticated use of raw base quality with the Logarithm Likelihood Ratio approach [16, 17]. Here, we propose a base recalibration tool to increase the sensitivity of minor allele detection while avoiding most sequence error hotspots. We also compare our base recalibration tool with others and discuss why they fail to accurately distinguish minor alleles from sequence errors.

We mainly compare our approach to the widely used GATK base recalibration [8]; Reqon [9] is another base recalibration tool, unfortunately, the severe memory demands of Reqon preclude use with vey high sequence coverage data. Some applications such as de novo genome assembly can also benefit from error correction tools (e.g. RACER [18]), but we exclude the comparison to such tools because they edit the bases directly instead of reducing the base quality scores, thereby precluding direct comparisons to our method. More importantly, such methods assume explicitly that there are sequence errors in reads contributing to low frequency kmers, which is not the case for true minor alleles of low level frequency. Our method also uses an additional training set spiked into the run (as do basecallers such as Ibis and Freeibis), however these basecallers recalibrate the raw fluorescence data to both reduce sequence errors and improve the accuracy in base quality, while our method recalibrates the measured base quality scores and thus affects the base quality only.

### Implementation

Sequencing error is often highly correlated with the machine cycle, observed nucleotide, nucleotide observed in the previous machine cycle, and read direction, and the goal of base recalibration is to remove systematic errors related to these conditions. Our approach, called SEGREG (for SEGmented REGression) works in two steps. In the first step, it divides bases in the spiked in training set (e.g. phiX174) into various groups, where a group is a combination of the following relevant conditions:

1. Whether it is the first or second read from paired-end reads
2. The machine cycle
3. The current observed nucleotide (A,C,G,T)
4. The observed nucleotide in the previous machine cycle

Segmented regression is then applied to each group according to the empirical base quality, which is calculated within the group by assuming all differences from the consensus sequence are sequence errors, excluding positions with known minor alleles. The segmented regression is given by:

$$ mi{n}_{a0,b0,a1,b1,bk}\left({\displaystyle \sum_{xr\_i<bk}}{w}_i*{\left(xe\_i-a0*xr\_i-b0\right)}^2+{\displaystyle \sum_{xr\_i\ge bk}}{w}_i*{\left(xe\_i-a1*xr\_i-b1\right)}^2\right) $$

Where y0 = a0*x + b0 and y1 = a1*x + b1 are two lines and bk is the break point, xr_i is the raw base quality, xe_i and w_i is the corresponding empirical score and the number of bases of xr_i respectively. For a typical pair ended library with read length 100, the bases in the training set are divided into 3200 groups (i.e. 2*100*4*4), and in some groups w_i can be small, which makes xe_i inaccurate (in the extreme, xe_i equals infinity if there is no error in w_i bases); we thus mask those groups with w_i < 100 from downstream analysis. As shown in Additional file 1: Figure S9, a number of bins (defined as per base quality score per group) have only a few bases, resulting in inaccurate estimates of empirical base quality, and hence these are masked. However, many bins have a sufficient number of bases for an accurate estimate of empirical base quality, especially for intermediate range of base quality scores which are of most interest (Additional file 1: Figure S9).

**Fig. 1:**
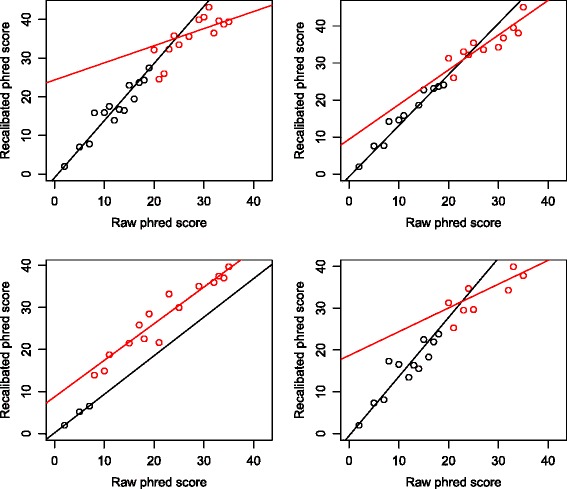
Four random examples of SEGREG recalibration. Bases are divided into groups based on the criteria described in the text, and in each group segmental regression is applied. The correspondence between raw and empirical phred scores is plotted as dots for bases of the same group and the breakpoint is chosen by the regression algorithm, with the two segments regressed to dots of the same color. If the dot (group) shown in the plot is based on less than 100 observations, the corresponding empirical base quality is imprecise and hence is not considered in the regression analysis

Figure 1 gives four examples of the results of segmented regression randomly selected from the thousands of groups. Segmented regression assumes that there is a linear relationship of raw quality score with the recalibrated score; we show below that assuming a more complicated relationship, such as a quadratic relationship, does not improve the performance of the method. We also assume that the background error rate of the basecaller is underestimated, which has a larger effect on higher base quality scores. For example, suppose there is a systematic error with error rate 0.001 underestimated by the sequencer. Then for the raw quality score of 40 (error rate 0.0001), the recalibrated score is 29.59 (total error rate 0.0011), while for the raw quality score of 10 (error rate 0.1), the recalibrated score is 9.96 (total error rate 0.101). In the second step, SEGREG once more assigns each base from the user’s data to one of these groups, e.g. j, and uses the bk_j and the regression lines y0_j and y1_j of the respective group to map the raw phred score to the recalibrated score. The source code is publicly available at https://github.com/sendru/SEGREG.

## Results and discussion

### Simulation

**Fig. 2:**
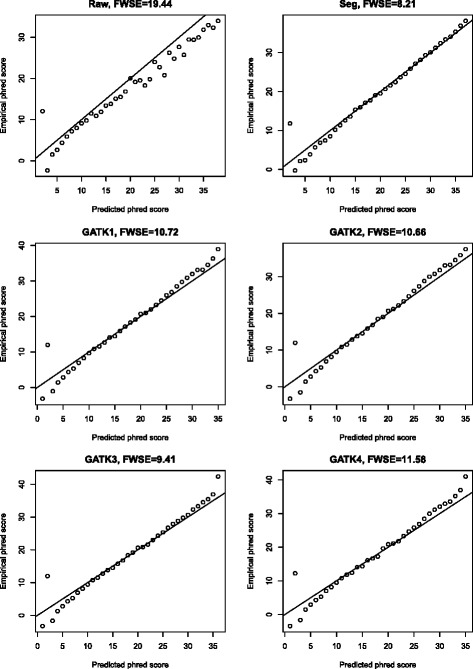
Recalibrated base quality scores generated from different methods for simulated data. For each method the recalibrated scores are compared to the empirical scores; the ideal (diagonal) line is shown in each plot. The Frequency-Weighted Squared Error (FWSE) is also given for each method. Raw: Sequence generated by simulation (SimSeq); Seg: this study; GATK1: dbsnp 142 is used as known database of SNPs, no misalignment issue (mapped by crossmap); GATK2: SNPs in the entire dataset of 13 mtDNA genome sequences, no misalignment issue; GATK3: the actual genetic variants (both SNPs and indels) for just the particular mtDNA genome sequence are used, no misalignment issue; GATK4: dbsnp142 is used, with potential misalignment issues (mapped by BWA)

We first use simulation data for the comparison of SEGREG with other approaches. Although any simulation is a simplification of real data, it has the advantage over real data in that all sequence errors and true minor alleles are known beforehand, whereas with real data it is not possible to distinguish all true minor alleles from sequence error hotspots, even in very high coverage data. For example, a common way of generating true minor alleles is to mix samples with different consensus sequences in different ratios and focus on the positions that differ in the consensus sequences as the only true minor alleles (e.g. [14]), but real samples may also contain true minor alleles which are then falsely considered to be sequence errors, thereby skewing the evaluation of the method. The simulated sequences were generated by Simseq. [19] In brief, thirteen complete mtDNA genome sequences representing the major haplogroups in modern humans were downloaded from NCBI and used as the templates for simulated reads (see Methods section for details); the sequence coverage is about 4000X. By pair-wise alignment between the revised Cambridge reference sequence (rCRS; reference) and the respective template sequences, all reads were aligned to the correct position in the rCRS by Crossmap [20] without misalignment issues. Additionally, we applied different training sets for GATK results: GATK1 used dbsnp142 [21] for known SNPs as the control; GATK2 used the polymorphic sites of all 13 template sequences as the control; GATK3 used the polymorphic sites of the respective template sequence (the actual genetic variants) as the control; and GATK4 used BWA [22] for alignment and dbsnp142 as the control, which represents a general use of GATK. We used the Frequency-Weighted Squared Error (FWSE) [9], which is defined as the sum of the squared errors between the predicted base quality and the empirical base quality, weighted by the relative frequency, to compare the accuracy of these tools. The recalibrated base quality in one of the simulations (with the template sequence belonging to haplogroup H1, which is of European origin) from different methods, as well as the raw quality scores (Bustard), are compared to the empirical scores in Fig. 2. In general, the FWSE is relatively high (compared to the FWSE from phiX174 in Additional file 2: Figure S6), because Simseq generates many bases with the lowest base quality (phred score = 2), which has a higher empirical base quality, and none of the methods can improve it. GATK4 has a larger FWSE than GATK1, which reflects the misalignment issue; with more knowledge of the actual genetic variants, GATK can improve the accuracy of recalibrated base quality as GATK2 has a lower FWSE than GATK1, and GATK3 has a lower FWSE than GATK2. Our method, SEGREG, has the lowest FWSE, which probably reflects both the direct regression on the multiple conditional probability in our model, as well as the simplicity of the error model generated from Simseq, which is based on mtDNA sequence data (Simseq reference).

**Fig. 3:**
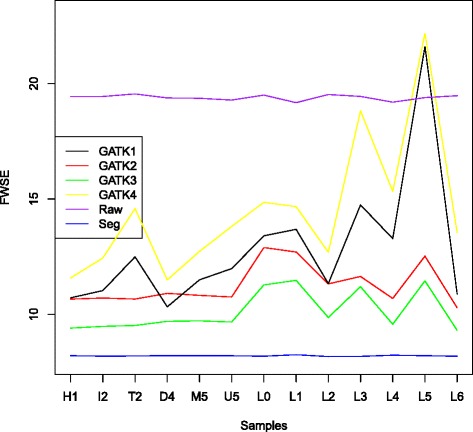
The FWSE for different base recalibration approaches applied to simulations based on 13 different mtDNA templates. The haplogroup for each mtDNA template is given on the X axis. The methods for comparison are the same as Fig. 2

Figure 3 gives the complete comparison of the 13 simulations. The relationships shown in Fig. 2 still hold in general with a few exceptions: In the template belong to haplogroup L5, there are 18 SNPs missing in dbsnp 142; as a result, both GATK1 and GATK4 has a larger FWSE value than the raw base quality, which means with an inappropriate database as the control, GATK can actually produce results that are worse than the raw base quality. GATK2 is not always better than GATK1, as shown for haplogroup D4, which together with the fact that GATK3 is always better than GATK2 reflects the impact of the choice of control SNPs on the results obtained with GATK. When compared to the FWSE from raw base quality and from SEGREG, GATK has more variance in all settings, especially for African haplogroups (haplogroups L0-L6), which may result from the fact that the reference genome (rCRS) belongs to haplogroup H2, which is mostly distributed in European populations.

In these simulations there are no true minor alleles in the data, i.e. any differences between reads and the consensus sequence are sequence errors. To simulate true minor alleles, we randomly selected a small portion of the reads from one template and mixed them with reads from another template, after correctly aligning them to the rCRS as described before. A total of 156 (13*12) pairwise mixture samples were generated with an average sequence coverage of 4000 and 0.3 % mixture, resulting in 9010 minor alleles as the true set. Note that not all the reads that differ from the consensus sequence at a true minor allele site will reflect true minor alleles. For example, suppose all bases have an error rate of 0.01, so the observed frequency of reads with different bases due to sequence error is 0.01/3 = 0.0033 (because there are a total of 4 bases). As a result, at a sequence coverage of 4000 we expect about 13 reads from sequence error and about 12 reads (=4000*0.03 %) from true minor alleles. We also need to consider the sampling variance, for example, with 0.3 % of reads randomly chosen from the second template, the actual mixture rate for all 9010 minor allele (Additional file 3: Figure S1) ranges from 0 to 0.72 %. In real applications, the variance should be even greater, given sequence error hotspots, PCR bias in GC rich regions and Illumina strand bias, etc.

Additional file 4: Figure S2 shows the distribution of FWSE in the simulated mixtures. The pattern is very similar to that for the previous simulation: GATK shows relative large variance and a few outliers. The SNP database used as the control for GATK does not appear to greatly influence the minor allele calling, as the frequency distribution of minor alleles that are included in dbsnp 142 does not differ from the distribution of minor alleles that are not in the database (Additional file 5: Figure S3).

**Fig. 4:**
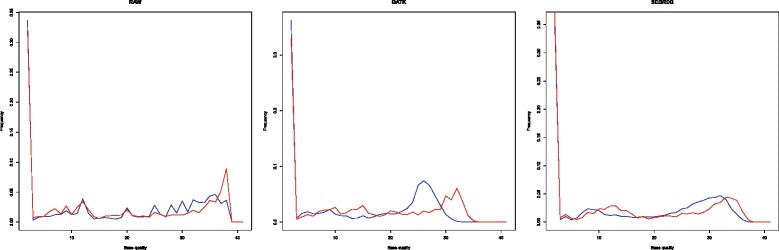
Frequency distribution of the base quality from each method. *Blue line*: the average frequency distribution of true minor alleles with the 1000 weakest signals; *red line*: the average frequency distribution of sequencing errors with the 1000 strongest signals (see main text for the explanation of weakest and strongest signals). Note that with GATK the weakest true minor alleles in general have lower recalibrated base quality scores than the strongest sequencing errors, while with SEGREG the distributions of recalibrated base quality scores are more similar for the weakest true minor alleles vs. the strongest sequencing errors

To see whether the recalibrated base quality provides an improvement in minor allele calling, we first assign every minor allele site a value, which can be written as ${\displaystyle \sum}\frac{x_i}{e_i}$, where x_i is the number of reads covering that site whose base quality score is i, and the e_i is the error rate corresponding to the phred score i. We then compared the 1000 lowest values among the 9010 true minor allele sites (i.e., true minor alleles receiving the weakest support) to the 1000 highest values among the remaining sites (i.e., sequence errors receiving the strongest support). The frequency distributions of the weakest true minor alleles and strongest sequence errors are shown in Fig. 4. As explained above, true minor allele sites are also covered by reads due to sequence errors, and a few additional reads from the true minor allele does not have a strong impact on the frequency distribution. However it is still surprising that GATK downgrades the base quality in the true minor allele sites. Apparently GATK assigns a lower base quality to minor alleles that are not at inferred SNP sites. Such a strategy is fine for SNP calling in reasonable sequence coverage, but it is detrimental for minor allele detection. Moreover, since reads from minor alleles constitute a very small portion of the total reads, the difference is not apparent in the FWSE. SEGREG, on the other hand, does not introduce such a bias and leaves the actual calling of SNPs to the downstream tools.

**Fig. 5:**
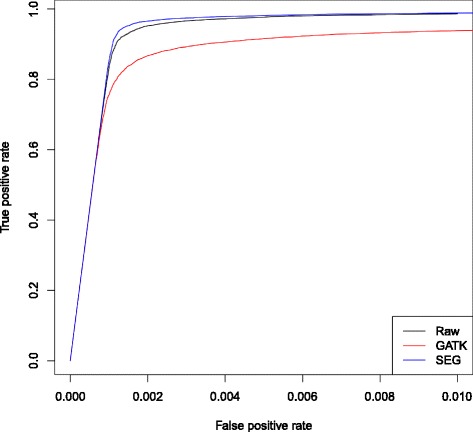
Receiver operating characteristic (ROC) curve for LLR from raw base quality (Bustard) and recalibrated base quality (GATK and SEGREG) scores

To give an objective evaluation of these tools, the logarithm likelihood ratio (LLR) [16, 17] was applied to the recalibrated score for minor allele detection. Briefly, LLR gives a likelihood ratio for each site; we then set cutoffs to the ratio as the classification model, which gives conditional positive (LLR > = c) and conditional negative (LLR < c) outcomes respectively. Formally, the true positive rate is defined as $\frac{{\displaystyle \sum } True\  positive}{{\displaystyle \sum } Conditional\  positive}$ and the false positive rate is $\frac{{\displaystyle \sum } False\  positive}{{\displaystyle \sum } Conditional\  negative}$. Using different cutoffs of the LLR, we then get the Receiver Operating Characteristic (ROC) curve of the different recalibration tools (Fig. 5). The fact that GATK has a smaller area under the ROC curve compared to the raw base quality confirms that GATK actually worsens the minor allele calling, even though it has a smaller FWSE.

We additionally used the simulation data for parameter tuning in applying LLR based-SEGREG to identify minor alleles. For the 9010 minor alleles produced in the artificial mixtures, the MAF estimated from raw read counts and that estimated by maximum likelihood are both highly correlated with the real MAF (Pearson correlation is 0.9988 for raw reads counts and 0.9941 for maximum likelihood). Additional file 6: Figure S4 shows the maximum LLR from sequence error at different sequence depths; increasing sequence depth results in higher raw quality-based LLR, but this pattern disappears after recalibration with SEGREG, indicating that SEGREG reduces this systematic bias. Moreover, a cutoff of LLR > 3 rules out almost all minor alleles due to sequence error. For a given site MAF, the LLR distribution for different sequence coverage is given in Additional file 7: Figure S5. In brief, for minor alleles with site MAF around 1 %, a sequence coverage of 1000 is needed for distinguishing such minor alleles from sequencing error; for site MAF around 0.3 % a sequence coverage of 3,000 is needed; and a sequence coverage > 16,000 is needed for site MAF around 0.1 %.

Although any simulation is a simplification of real data, there are a number of insights that arise from knowing the true minor alleles vs. the sequence errors. First, for a given minor allele frequency there can still be substantial variation in the number of covered reads with the minor allele (Additional file 3: Figure S1), which can contribute to difficulties in distinguishing true minor alleles from sequence errors. Second, even with a constant observed MAF, LLR separates true minor alleles from sequence errors at different rates depending on the sequence coverage. Third, the maximum LLR (without recalibration) from sequence error increases with the sequence coverage (Additional file 6: Figure S4). These observations would not arise from real data, where true minor alleles cannot be distinguished with certainty from sequence errors.

### Real sequence data

We next used phiX174 DNA (from phage cultured in our institute) for comparison. Briefly, reads from spiked-in phiX174 in one Illumina Hiseq2500 run were extracted, resulting in a total of 3.77 million reads without PCR duplication and an average coverage of about 87,500. Two previously reported polymorphic sites (positions 1401 and 1644) [7, 23] were also detected and masked from the downstream analysis. The consensus sequence was called and used as the reference to control for misalignment issues. The result of GATK and SEGREG are shown in Additional file 2: Figure S6, along with results for SEGREG where we only used half of the data for the training set. All three methods improve the base quality to high accuracy; however note that GATK implementation is atypical. First, it does not have the misalignment issue; and second, all of the occurring genetic variants are included in the control SNP database (equivalent to GATK3 in Fig. 2). Note also that using half the data for the training set produces acceptable results, although the performance of SEGREG improves with more reads in the training set (Additional file 2: Figure S6).

We also applied quadratic regression to the phiX174 data (Additional file 8: Figure S10) to obtain recalibrated base quality scores, however quadratic regression does not provide an overall improvement. Part of the reason is that there are only a few bases in many bins (Additional file 1: Figure S9), which makes the empirical base quality inaccurate. Moreover, the overall linear relationship between the recalibrated and the raw base quality also appears to provide important information.

A common assumption used in general SNP calling pipelines is that all low frequency differences from the consensus sequence are sequence errors. Any application that explicitly (such as RACER for error correction) or implicitly (such as GATK base recalibration) uses this rule will be biased against detecting true minor alleles. We also show that FWSE is not an appropriate measure for comparing different methods, since reads from the minor alleles constitute a very small portion of the total reads, and hence the best way to compare different measures is using the ROC curve, which in turn requires knowing the true minor alleles.

**Fig. 6:**
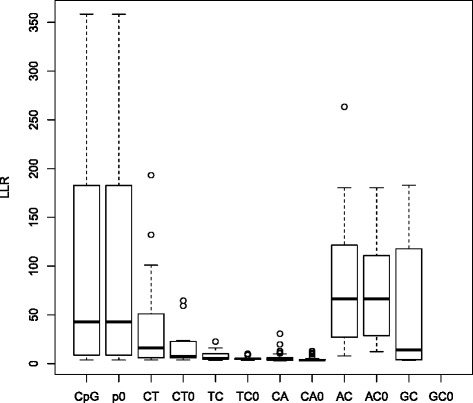
Boxplot of the LLR distribution for different types of minor alleles (LLR > 3) observed in 6 runs of phiX174. Results from the strand bias and position rank sum tests are also shown, as follows, with the number of alleles in parentheses: CpG(15), minor alleles on CpG sites; p0(15), CpG after SB < 1 and PosRankSum > -3 filters; CT(20), C- > T mutations excluding CpG sites, where T is the minor allele; CT0(16), CT after filters; TC(14), T- > C mutations; TC0(10), TC after filters; CA(137), C- > A mutations; CA0(36), CA after filters; AC(26), A- > C mutations; AC0(24), AC after filters; GC(7), G- > C mutations; GC0(0), GC after filters. No A- > T minor alleles were observed

The phiX174 phage is cultured from a single strain, and thus in many applications any differences between reads and the consensus are assumed to be sequence errors. However, as found both in our study and previously [23], true minor alleles exist with a frequency around 25 % at positions 1401 and 1644. To further investigate whether there are additional true minor alleles, we applied SEGREG independently to 7 runs of phiX174 sequences (data can be retrieved from EMBL: PRJEB11001, where the training set and the user’s data are the same) from different Illumina platforms (Hiseq2500 and Miseq) and different sequence callers and analyzed them with the LLR approach. Overall, the true minor alleles are expected to be observed in different runs, although weak signals might not be detected in every run. The observed minor alleles with LLR > 3 were further divided into several groups: CpG site (CpG); C- > T mutation (or G- > A on the complement) where C is the major allele and T is the minor allele except CpG sites; T- > C mutation (TC); C- > A mutation (CA); A- > C mutation (AC); and G- > C mutation (GC); T- > A mutations were not considered as none were observed. Additionally, we applied a strand bias test [24] and a Mann-Whitney U test on the position within the read, called PosRankSum, to these minor alleles, requiring SB < 1 (corresponding to a *p*-value <0.01) and PosRankSum > -3 (corresponding to a *p*-value of 0.003). Minor alleles in CpG sites are the only group whose minor alleles all passed these two tests, suggesting they are true minor alleles, while C- > A mutations decreased from 137 to 36 and G- > C mutations from 7 to 0 after applying these filters, indicating they are caused by sequencing errors. The large quantity of sequence errors in C- > A mutations also means that the Illumina chemistry has a high error rate in distinguishing A from C, which might explain the fact that A- > C mutations have a relative large LLR score and are almost unchanged after additional tests (from 26 to 24), suggesting it is the main source for sequence error hotspots. The LLR distribution for each group of minor alleles is shown in Fig. 6.

Although strand bias test and position rank sum test are effective to remove false minor alleles, the resulting minor alleles after filtering still have a low transition to transversion ratio (0.68), indicating that most of them are still sequence error hotspots. In Additional file 9: Table S1, we additionally set the cut-off to >0.001 in MAF and compared minor alleles in these runs. The strand bias test and position rank sum test look reasonable since minor alleles filtered by these tests are sequencer/basecaller specific, and concentrated in the region 3012–3035 near two GGT motifs (3021 and 3033), which is reported to be an error prone pattern [25]. Four minor alleles from AC mutations are likely to be sequence error hotspots as discussed before. Besides the two previous reported minor alleles (1401 and 1644), we additionally report two sites that by our criteria harbor true minor alleles (878 and 5349). The only position that is unclear is position 2339, but its MAF is very close to the cutoff (0.001). Although these two additional suggested minor alleles are likely to be true minor alleles, overall there are too few minor alleles in the phiX174 data to give a meaningful ROC curve.

We next applied SEGREG-based LLR to 247 mtDNA genome sequences [26], and compared our results to a previous LLR pipeline [17]. With mtDNA, the transition to transversion (Ti/Tv) ratio can also be used as an indicator of error rate for large datasets, as mtDNA mutations show a strong excess of transitions over transversions [27]. The sequencing coverage of the 247 mtDNA samples ranges from 693 to 34180, with median sequence coverage of 2809. We found 2164 minor alleles with a Ti/Tv ratio of 14.80 from the previous LLR pipeline [17] (i.e. MAF > 1 % and at least 2 reads from the minor allele from both strands, LLR > 5). Using SEGREG-based LLR as shown in Additional file 10: Figure S7, we found 32929 minor alleles with Ti/Tv ratio 14.89, which means we found 14 times more minor alleles while keeping the error rate at the same level. Some of this discrepancy is due to the fact that the previous pipeline uses additional criteria to identify true heteroplasmies vs. potential contamination and nuclear inserts of mtDNA (nuMTs), whereas our pipeline only tries to distinguish true minor alleles (regardless of cause) from sequence errors. However, if we take the same number of minor alleles (2164) with the highest LLR, the Ti/Tv ratio is above 30 (Additional file 10: Figure S7). Since heteroplasmies represent ongoing mutation and the Ti/Tv ratio declines when sequences from longer evolutionary distances are compared [28], the apparent two levels of Ti/Tv ratio in Additional file 10: Figure S7 might reflect heteroplasmies and contamination/nuMT effects respectively. To further validate that the Ti/Tv ratio is meaningful in this context, Additional file 11: Figure S8 shows the Ti/Tv distribution among these 247 samples for inferred true minor alleles vs. sequence errors.

## Conclusions

Detection of minor alleles with low frequency in very high coverage (>1000X) sequence data is still a challenging task, mainly because both a good model for sequence errors and an accepted “gold standard” for calling true minor alleles with low level frequency in real sequence data are lacking. Despite these limitations, our method SEGREG offers several improvements compared to GATK. First, the GATK BaseRecalibration modulo requires the IndelRealignment modulo to run properly for the user’s data, which is often computationally infeasible for very high sequence coverage and thus introduces additional bias. By contrast, SEGREG merely requires a training set (e.g. phiX174) to align correctly, which is relatively fast and easy to achieve by mapping reads to the consensus sequence. Second, as we shown in the simulations (Figs. 2 and 3), the performance of GATK depends greatly on the control database of known SNPs, and in many applications there will be additional SNPs in the data. SEGREG, on the other hand, only requires the genetic variants of the spiked-in control sequence, (i.e. with phiX174, positions 1401, 1644, 878 and 5349 would be excluded from the empirical base quality calculation (Additional file 9: Table S1)). While it is likely that there are additional true minor alleles in phiX174, especially in CpG sites, (Fig. 6), the very low MAF for these sites (MAF < 0.001), means they introduce little bias in the empirical base quality calculation. Lastly, but most importantly, the conditional error model in GATK can be represented by

$$ \Pr \left(\mathrm{e}\Big|{\mathrm{a}}_1,\dots, \mathrm{a}\_\mathrm{n}\right)={\displaystyle \sum }{\mathrm{cov}}_{\mathrm{i}}* \Pr \left({\mathrm{e}\Big|\mathrm{a}}_{\mathrm{i}}\right) $$

Where a_i are conditions used in the model, and cov_i are covariant functions for each condition. GATK estimates Pr(e|a_i) independently and uses machine learning for covariants, while SEGREG estimates Pr(e|a_1,…,a_n) directly from the training data. While SEGREG requires more reads in the training set than GATK, as the performance is decreased if SEGREG is trained on only half of the reads (Additional file 2: Figure S6), nevertheless SEGREG exhibits greatly improved performance in minor allele detection.

We also show that FWSE is not appropriate for evaluating different base quality recalibration tools in terms of minor allele detection, since some tools such as GATK improve the overall precision of SNP calling at the cost of failing to detect low frequency minor alleles. However, a thorough comparison of SEGREG and other base quality recalibration tools would need true minor alleles in real sequence data; in the absence of such data, we relied on simulation data for such comparison. By comparing the SEGREG-based LLR results from different runs of phiX174 DNA, minor alleles that are likely to be true minor alleles, such as those at CpG sites due to the elevated mutation rate at such sites, are mixed with minor alleles that are likely to be sequence error hotspots in Illumina reads, such as A- > C mutations. Since repeated runs for a single strain such as phiX174 has mixed signals from both true minor alleles and sequence error hostspots, distinguishing these remains a challenge for further studies.

Nevertheless, our method SEGREG does improve detection of mtDNA heteroplasmies. A commonly-used approach for detecting such minor alleles is to set a cutoff in MAF to a high level and concentrate on significant minor alleles only, thereby missing true minor alleles with low MAF. By using the Ti/Tv ratio as an additional error indicator for mtDNA, our results for MAF > 0.1 % shows the same level of error rate as MAF > 1 % from the previous pipeline [17]. This improvement is due to the improved performance of SEGREG-based LLR (as shown in the simulation data). Additionally, the Ti/Tv ratio (Additional file 2: Figure S6) provides useful information in distinguishing different sources of minor alleles in mtDNA. Overall, SEGREG-based LLR provides investigators with a new and more accurate approach for identifying true minor alleles in high coverage sequence data.

## Methods

### Logarithm Likelihood Ratio (LLR)

The likelihood function is defined as:

*L*(*f*) = ∏_*j* = 1_^*l*^[(1 − *f*)*ε*_*j*_ + *f*(1 − *ε*_*j*_)]∏_*j* = 1_^*k*^[(1 − *f*)(1 − *ε*_*j*_) + *fε*_*j*_]

where epsilon is the error rate derived from the phred score, f is the minor allele frequency and l and k are the number of reads covering the minor and major allele respectively (without loss of generality, only minor alleles with the most reads covered are considered). Maximum likelihood is used to estimate the minor allele frequency $\widehat{f}$, and the LLR is then calculated as $\log \left(L\left(\widehat{f}\right)/L(0)\right)$, which can be interpreted as the relative likelihood that MAF= $\widehat{f}$ vs. MAF = 0.

### Simulated data

SimSeq (https://github.com/jstjohn/SimSeq) was used to produce the simulated data. The sample error profile in the same package was used as the error model. Thirteen complete human mtDNA genomes representing major haplogroups were downloaded from NCBI (genebank KC911603.1, KJ786931.1, KJ786932.1, DQ304903.1, KF179062.1, KC911354.1, KC911353.1, JN580306.1, KC911364.1, HQ873516.1, KC911596.1, KJ756350.1, KJ801919.1) and used as the templates, with no other parameters specified. To generate reads evenly distributed across the circular mtDNA genome, the first 1000 bp was added to the end of the rCRS sequence; 4 million reads were generated for each template and reads completely within the artificial repeat region were discarded. Crossmap version 0.2.1 was used to align the reads to the reference (rCRS) to remove alignment artefacts. The total sequence coverage was about 40,000 as the library for each template. Pairwise mixtures were then performed by randomly taking 10 % of the reads from one library and mixing them with 0.03 % of the reads from the other. dbsnp 142 was used as an additional training set for GATK, while SEGREG takes one of the libraries as the training set for parameter tuning, and applies the results to all of the other libraries. Minor alleles were obtained by the pairwise alignment tool Emboss Strecher [29]; a total of 9034 putative minor alleles were detected. Removing those within 5 bp of an indel resulted in a total of 9010 minor alleles for further analysis.

### Mapping reads to the mtDNA genome

Network-aware BWA (https://github.com/udo-stenzel/network-aware-bwa), which is equivalent to BWA 0.5.10, was used for mapping. An in house program equivalent to MIA [30] was used for calling the consensus mtDNA sequences. All reads were then mapped to the consensus sequence with the first 1000 bp added to the end, and only proper reads defined by BWA (extracted by samtools –f 0x3) [22] were used in this study with no cut-off set for mapping quality. Those reads with both segments completely in the artificial repeat region were moved to the corresponding position in the first 1000 bp by a custom C++ program, followed by duplication removal with Picard (https://github.com/broadinstitute/picard). This strategy maps reads covering the whole mitochondrial genome with minimum sequence gaps, although reads from nuclear mitochondrial sequence (nuMTs) may be wrongly aligned to mtDNA. By contrast, mapping reads first to the complete human genome and keeping only those reads with mapq > = 20 will reduce nuMT influence but will also produce reduced coverage and even gaps for the authentic mtDNA genome, due to incorrect assignment of mtDNA reads that overlap with nuMTs. As in this study we are only concerned with distinguishing true minor alleles from sequence error, rather than distinguishing different causes of minor alleles (e.g. contamination, nuMTs, etc.), a strategy that maximizes mtDNA coverage rather than minimizing nuMT reads is preferred.

### PhiX174

The phiX174 DNA was obtained from Life Technologies and cultured further in house. It has 6 known SNPs compared to the reference (NCBI_001422.1) and no PCR is involved in the preparation of the phiX174 DNA. The phiX174 was used as a control and the training set in Illumina multiplex sequencing, and various Illumina platforms (Hiseq2500 and Miseq) as well as various sequence callers (Bustard, Ibis, Freeibis) were used. The two Miseq runs were merged for comparable sequence coverage. By using the same strategy as with mtDNA sequences (except for duplication removal), we mapped reads to the phiX174 genome with sequence depth ranging from 77,000 to 125,000 for the Hiseq2500 and 18,000 for the Miseq platform (Additional file 9: Table S1).

### Heteroplasmy calling in mtDNA

The following filters were used to identify mtDNA heteroplasmies: bases with phred score <20 were not counted; at least 2 reads with the minor allele from each strand were required; and the LLR cutoff was set to 3 after SEGREG base recalibration. We also required a minimum 0.1 % MAF (estimated from maximum likelihood), a strand bias ratio < 1 [24], and a Mann–Whitney U test on read position (PosRankSum) > -3 (Additional file 3: Figure S1). Minor alleles in repetitive regions of the mtDNA genome that are prone to misalignment and frequent indels (302–316, 513–526, 566–573 and 16181–16194) were excluded from the analyses.

### Availability of supporting data

The SEGREG program is publicly available at https://github.com/sendru/SEGREG

The raw sequencing data for phiX174 is publicly available at EMBL: PRJEB11001

## Additional files

Additional file 1: Figure S9. The distribution of the number of bases in each bin, which is defined as per phred score (typically ranging from 2 to 41) per group. Left, frequency distribution of the number of bases per bin. Right, the number of omitted bins (with <100 bases) plotted against base quality score, showing that bins are mostly omitted for very low or very high base quality scores. (PDF 13 kb) Additional file 2: Figure S6. Recalibrated base quality scores for simulated data analyzed with different methods, compared to the empirical score. The ideal (diagonal) line is shown in each plot. The Frequency-Weighted Squared Error (FWSE) is given for each method. Raw: Illumina default sequencer; GATK: Mapping to the consensus sequence and the known minor alleles (MAF > 5 %) are used as the known SNPs. Seg: this study; Seg_half: the same as Seg but with half of the reads chosen randomly for the training set. (PDF 6 kb) Additional file 3: Figure S1. The site MAF distribution for 9010 minor alleles created by artificial mixture with a mean mixture of 0.3 % and a sequence coverage of 4000. The variation is caused by the random distribution of reads along the genome. (PDF 4 kb) Additional file 4: Figure S2. Boxplot of FWSE distributions based on 156 simulated mixed datasets analyzed by different methods. Raw: the simulation data; GATK: using db142 as the control with no misalignment issue (corresponding to GATK1 in Fig. 2); SEGREG: this study. (PDF 4 kb) Additional file 5: Figure S3. The frequency distribution of GATK (with dbsnp142 as the control) recalibrated base quality. Black: sequence error; Red: SNPs in dbsnp 142; Blue: SNPs not found in dbsnp 142. (PDF 4 kb) Additional file 6: Figure S4. The maximum LLR from sequence error in simulated data under different sequence depths. For the raw data increasing sequence dept results in increased LLR from sequence errors, whereas after SEGREG base recalibration, increasing the sequence depth results in lower LLR. (PDF 11 kb) Additional file 7: Figure S5. The sequence depth dependency for minor allele detection in SEGREG-based LLR. Minor alleles with different average levels of MAF (0.1 %, 0.3 % and 1 %) were tested in simulations in each plot. LLR > 3 (from Additional file Figure S4) is used to distinguish true minor alleles from sequence error. (PDF 97 kb) Additional file 8: Figure S10. A comparison of segmented vs. quadratic regression applied to base score recalibration for phiX174 data. Quadratic regression does not result in an overall improvement. (PDF 5 kb) Additional file 9: Table S1. Fifteen minor alleles reported from analysis of phiX174 by various sequence callers. For each analysis, the minor allele is reported only if LLR > 3, otherwise the entry is FAIL. We also omit minor alleles with MAF < 0.1 % in all analyses. The results of the Strand bias test (SB <1) and Position rank sum test (Pos_rank_sum > -3) are also reported. The sequence coverage for each analysis is in the last row of the table. Further notes about the nature of the minor allele are provided in the last column of the table. (PDF 45 kb) Additional file 10: Figure S7. The cumulative Ti/Tv ratio for minor alleles in mtDNA, ordered according to decreasing LLR. The first 3,000 minor alleles have a Ti/Tv >30, then the ratio decreases to and stabilizes around 18 for the next 5,000 to 30,000 minor alleles. The high Ti/Tv ratio suggests these minor alleles are probably true but have different sources: those with the highest Ti/Tv ratio are from heteroplasmy, which reflects recent mutations, while the rest probably stem from cross contamination or nuMTs, which diverged from the major allele thousands to millions years ago and hence would have a relatively lower Ti/Tv ratio. (PDF 125 kb) Additional file 11: Figure S8. Ti/Tv ratio among 247 mtDNA samples for minor alleles covered by at least two reads from each strand. Positive set: minor alleles with Segreg based LLR > 3, MAF > 0.001, SB < 1, PosRankSum > -3. Negative set, all other minor alleles. (PDF 4 kb)

## Abbreviations

Bp: basepair
LLR: logarithm likelihood ratio
MAF: minor allele frequency
mapq: mapping quality
mtDNA: mitochondrial DNA
nuMTs: nuclear mitochondrial sequence
PDF: probability distribution function
rCRS: revised Cambridge reference sequence
Ti/Tv: transition to transversion
