# Supplementary figures and images for: Improvement in detection of minor alleles in next generation sequencing by base quality recalibration
Source: BMC Genomics. 2016 Feb 27;17:139. doi: 10.1186/s12864-016-2463-2 (PMC4769523; doi:10.1186/s12864-016-2463-2)

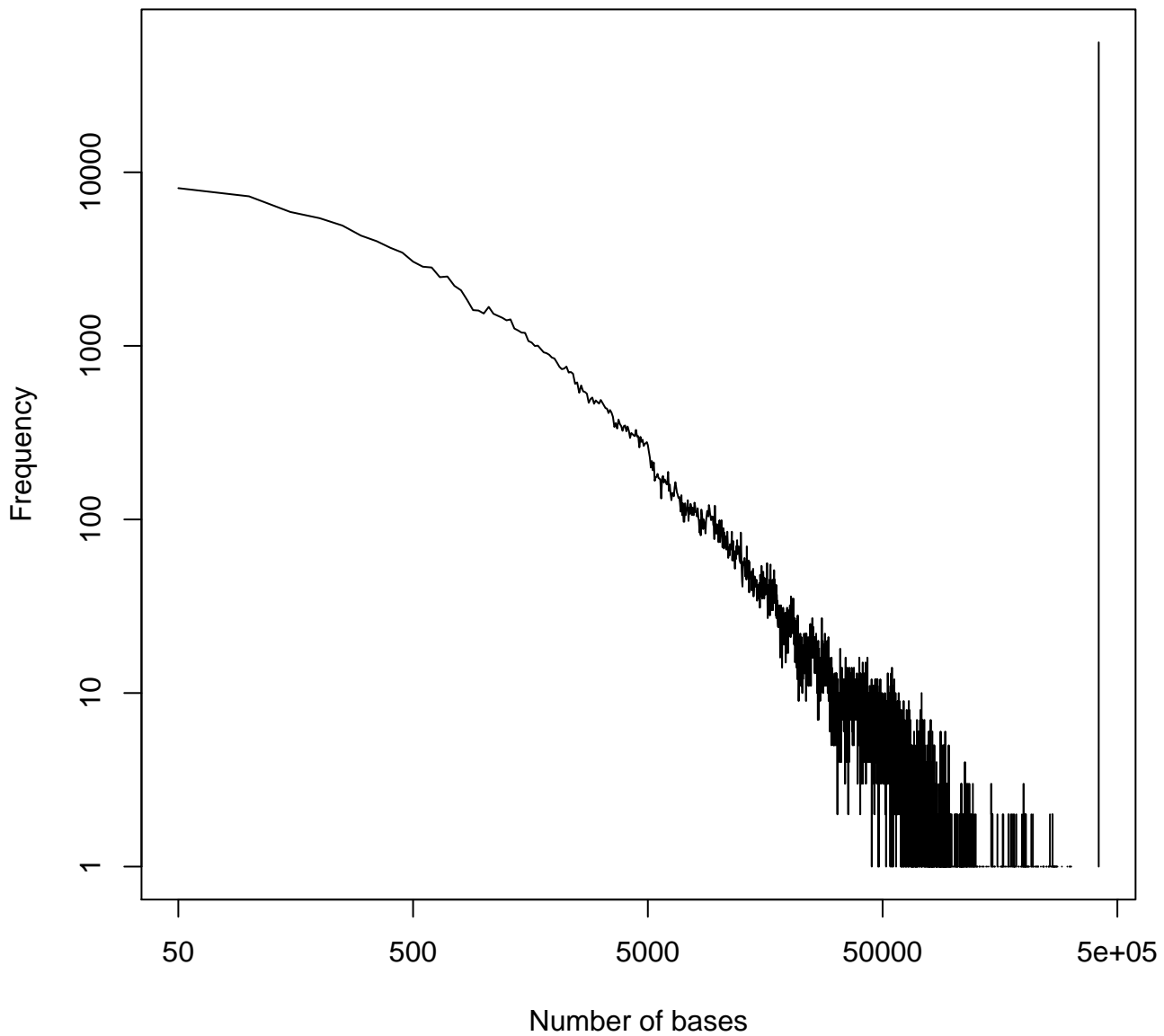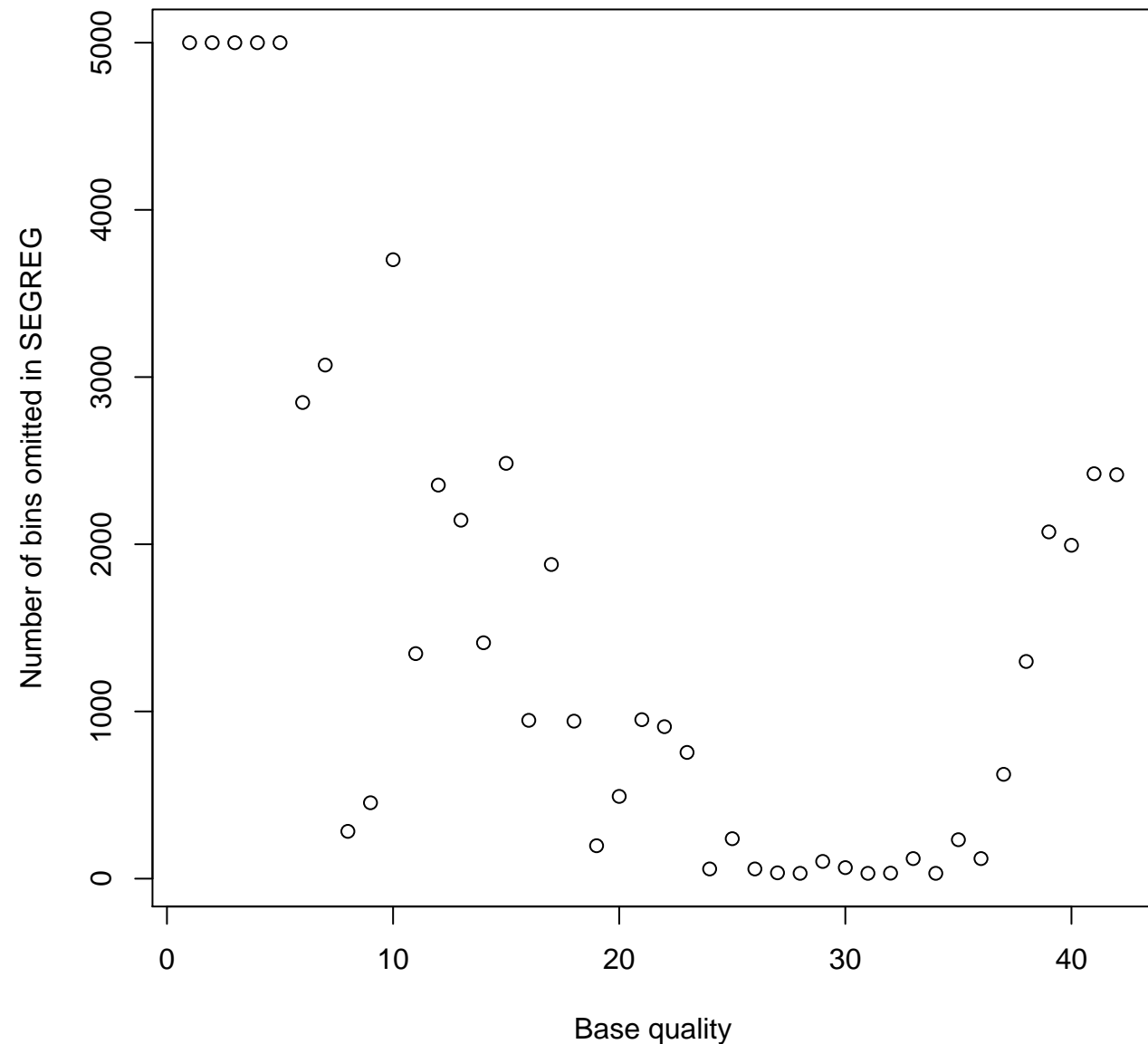

Supplement: Additional file 1: Figure S9. — The distribution of the number of bases in each bin, which is defined as per phred score (typically ranging from 2 to 41) per group. Left, frequency distribution of the number of bases per bin. Right, the number of omitted bins (with <100 bases) plotted against base quality score, showing that bins are mostly omitted for very low or very high base quality scores. (PDF 13 kb) [file 12864_2016_2463_MOESM1_ESM.pdf]

**Raw, FWSE=11.75**

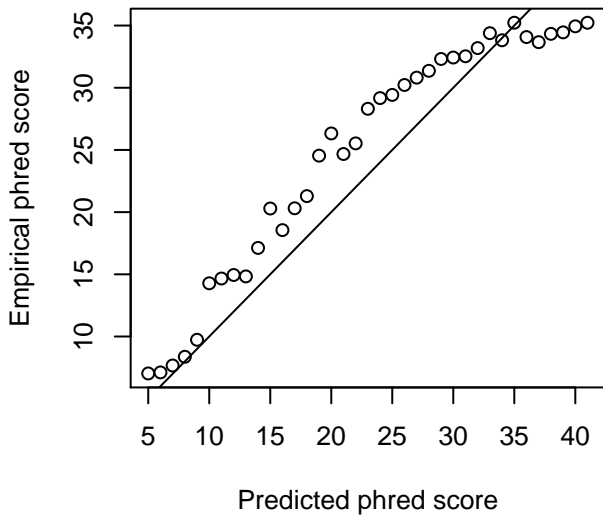

**GATK, FWSE=0.63**

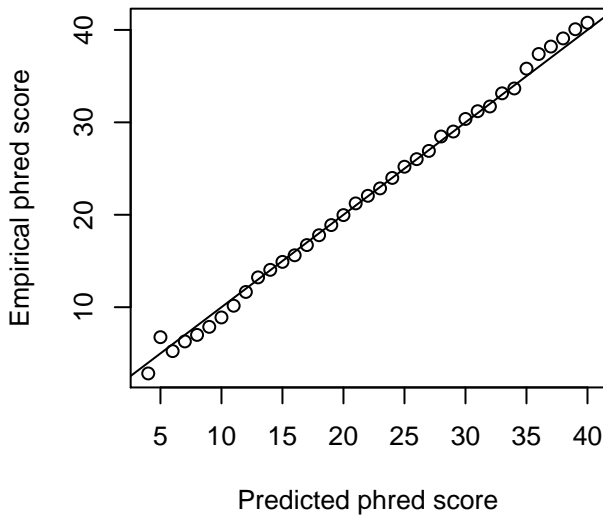

**Seg, FWSE=0.25**

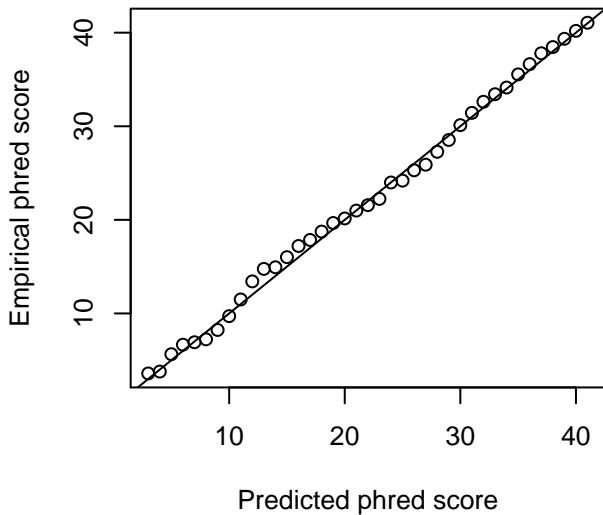

**Seg\_half, FWSE=1.42**

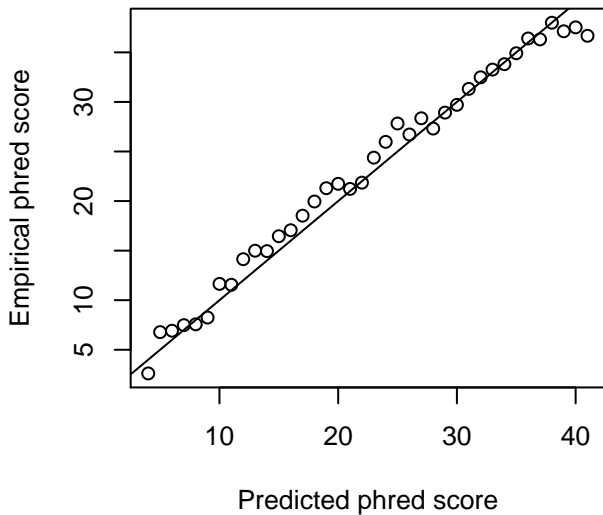

Supplement: Additional file 2: Figure S6. — Recalibrated base quality scores for simulated data analyzed with different methods, compared to the empirical score. The ideal (diagonal) line is shown in each plot. The Frequency-Weighted Squared Error (FWSE) is given for each method. Raw: Illumina default sequencer; GATK: Mapping to the consensus sequence and the known minor alleles (MAF > 5 %) are used as the known SNPs. Seg: this study; Seg_half: the same as Seg but with half of the reads chosen randomly for the training set. (PDF 6 kb) [file 12864_2016_2463_MOESM2_ESM.pdf]

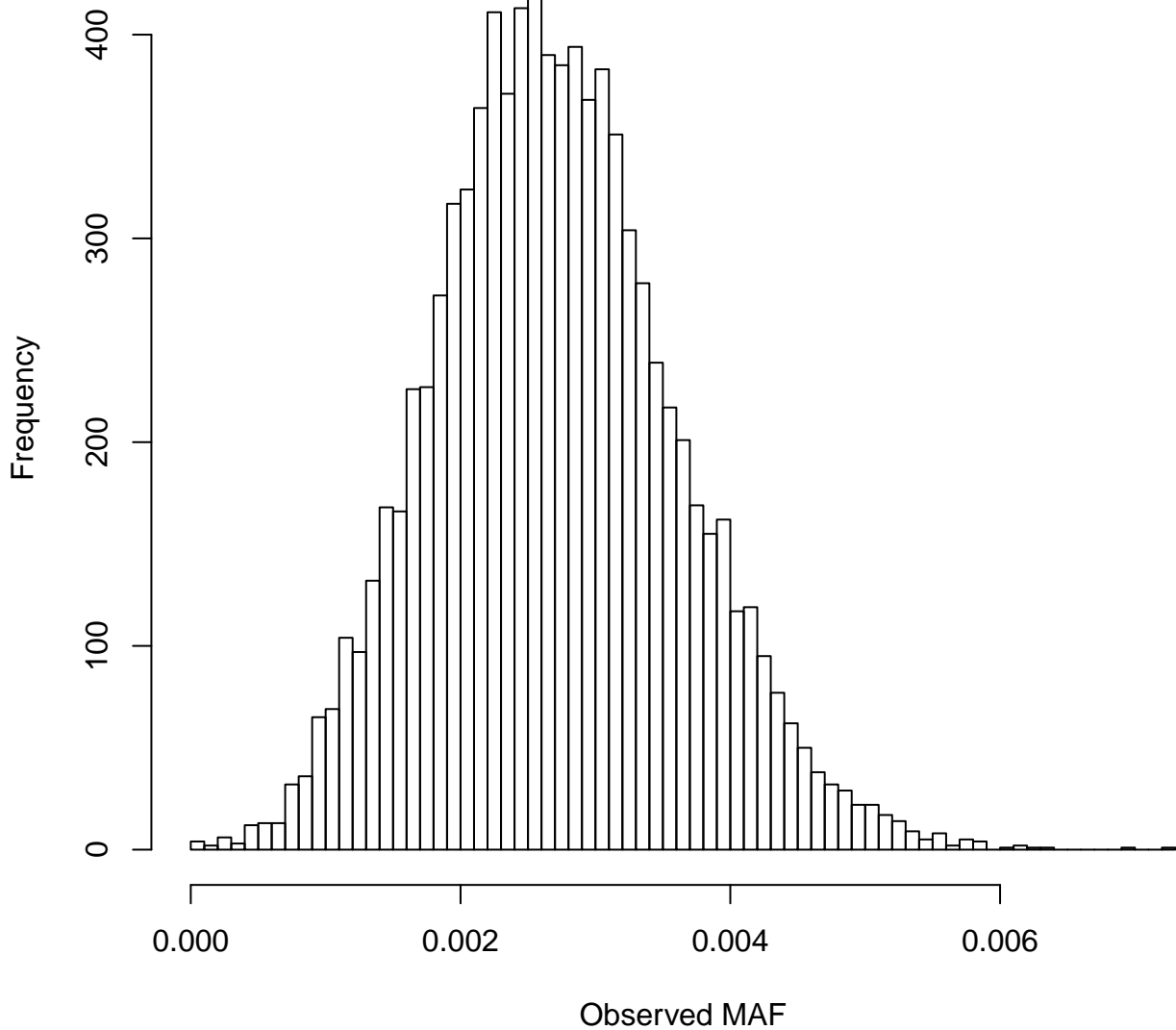

Supplement: Additional file 3: Figure S1. — The site MAF distribution for 9010 minor alleles created by artificial mixture with a mean mixture of 0.3 % and a sequence coverage of 4000. The variation is caused by the random distribution of reads along the genome. (PDF 4 kb) [file 12864_2016_2463_MOESM3_ESM.pdf]

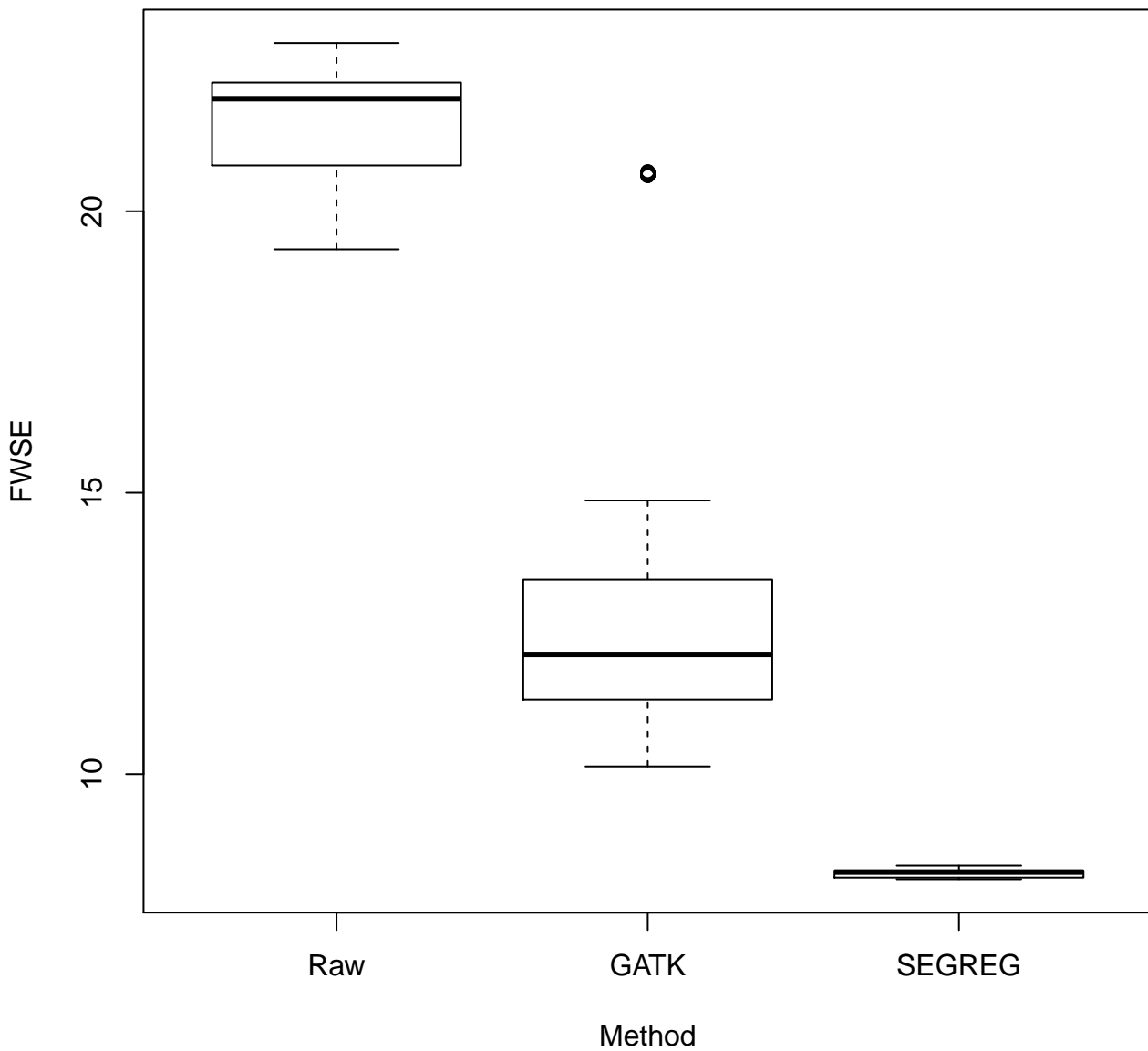

Supplement: Additional file 4: Figure S2. — Boxplot of FWSE distributions based on 156 simulated mixed datasets analyzed by different methods. Raw: the simulation data; GATK: using db142 as the control with no misalignment issue (corresponding to GATK1 in Fig. 2); SEGREG: this study. (PDF 4 kb) [file 12864_2016_2463_MOESM4_ESM.pdf]

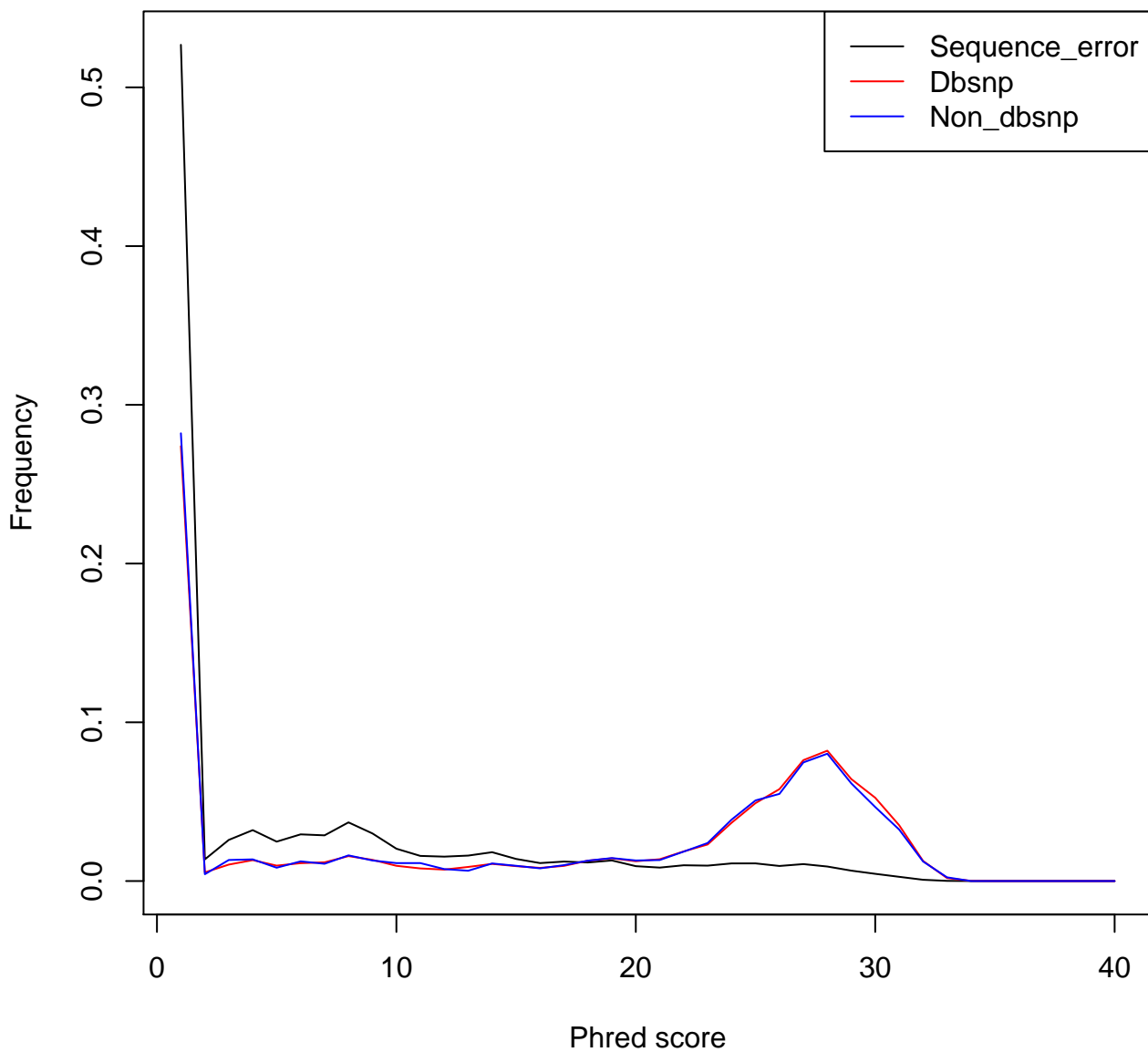

Supplement: Additional file 5: Figure S3. — The frequency distribution of GATK (with dbsnp142 as the control) recalibrated base quality. Black: sequence error; Red: SNPs in dbsnp 142; Blue: SNPs not found in dbsnp 142. (PDF 4 kb) [file 12864_2016_2463_MOESM5_ESM.pdf]

**The maximum LLR from sequence error(RAW)**

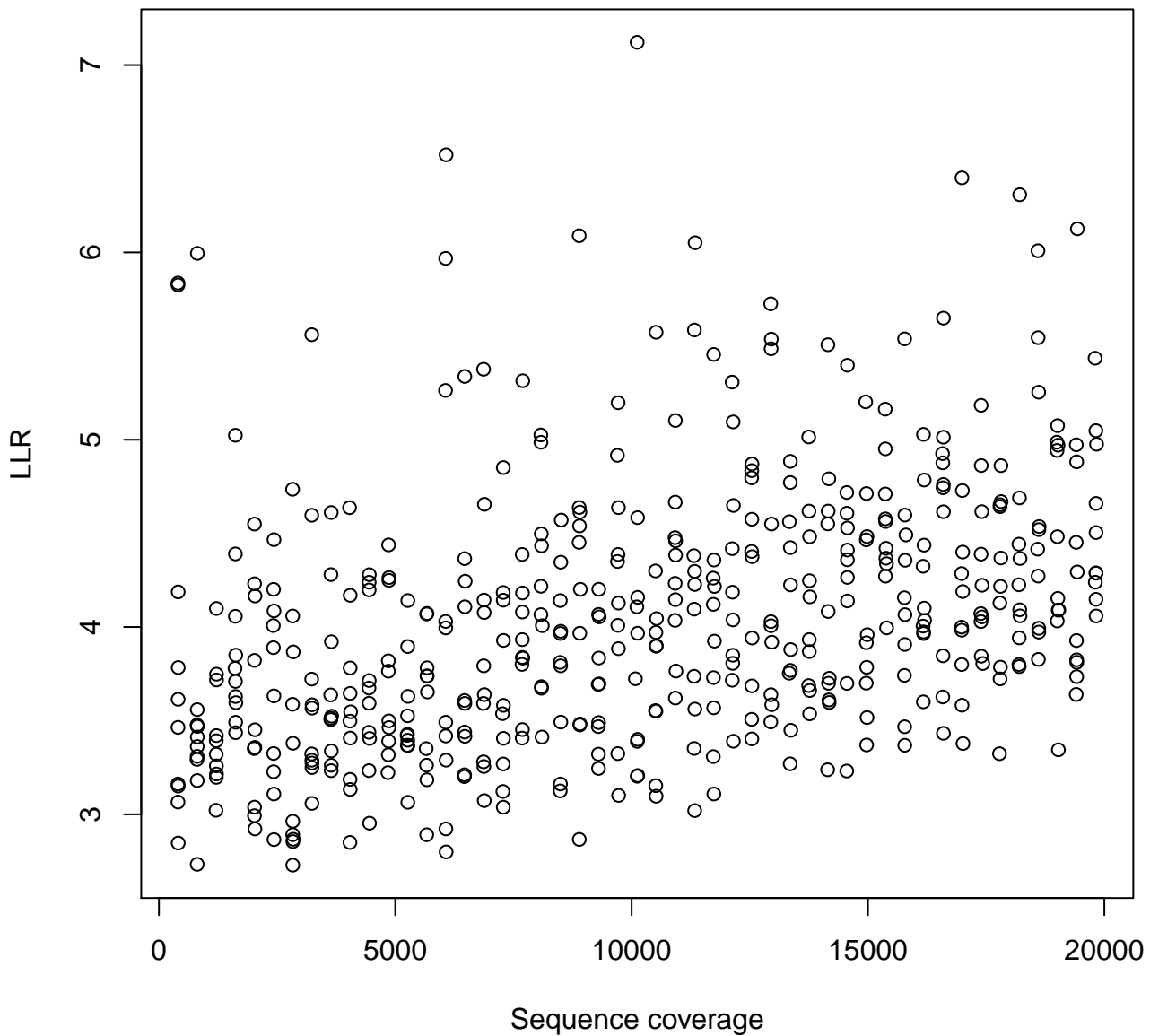

**The maximum LLR from sequence error(SEGREG)**

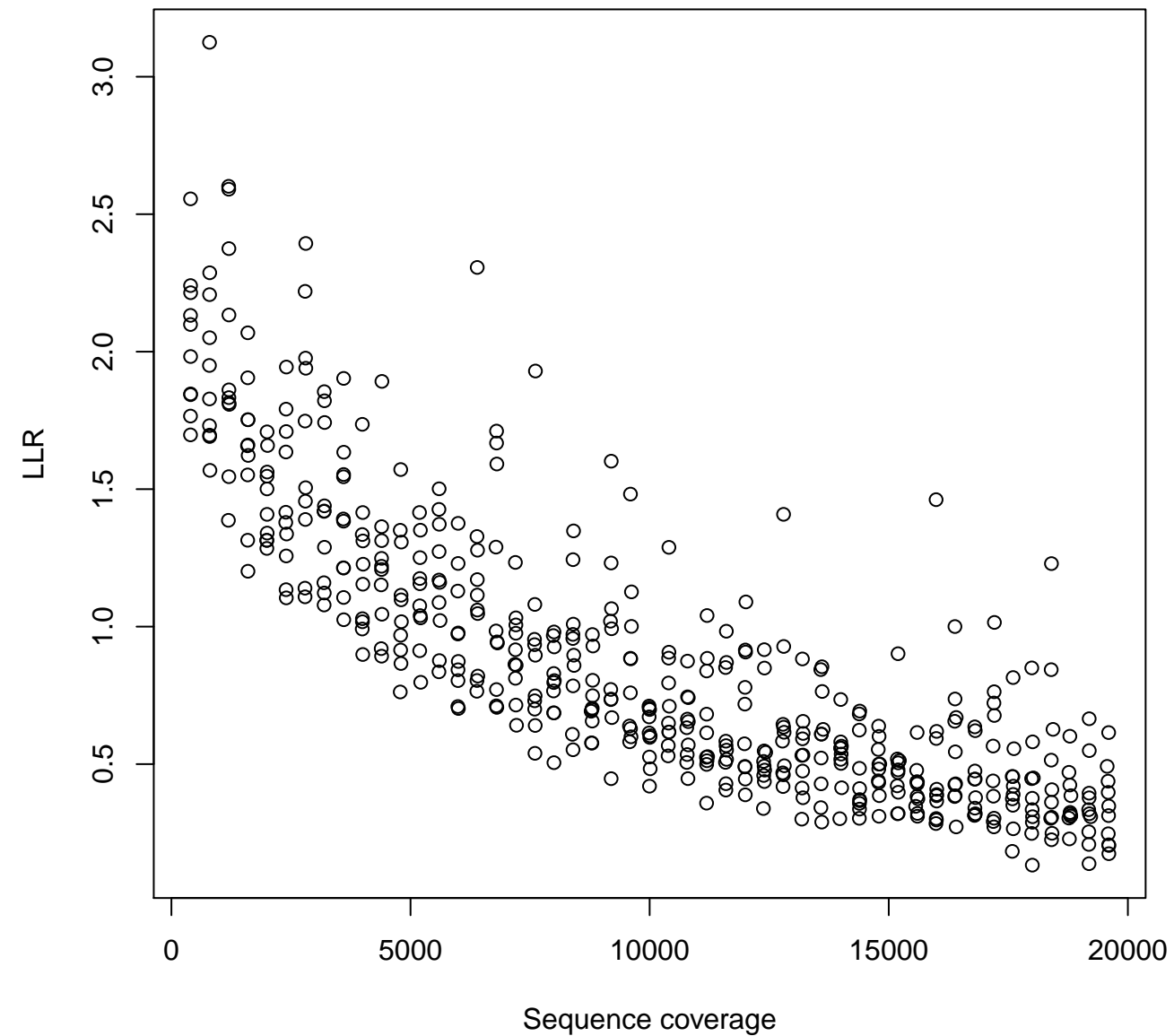

Supplement: Additional file 6: Figure S4. — The maximum LLR from sequence error in simulated data under different sequence depths. For the raw data increasing sequence dept results in increased LLR from sequence errors, whereas after SEGREG base recalibration, increasing the sequence depth results in lower LLR. (PDF 11 kb) [file 12864_2016_2463_MOESM6_ESM.pdf]

Simulation of site MAF 0.098%~0.102%

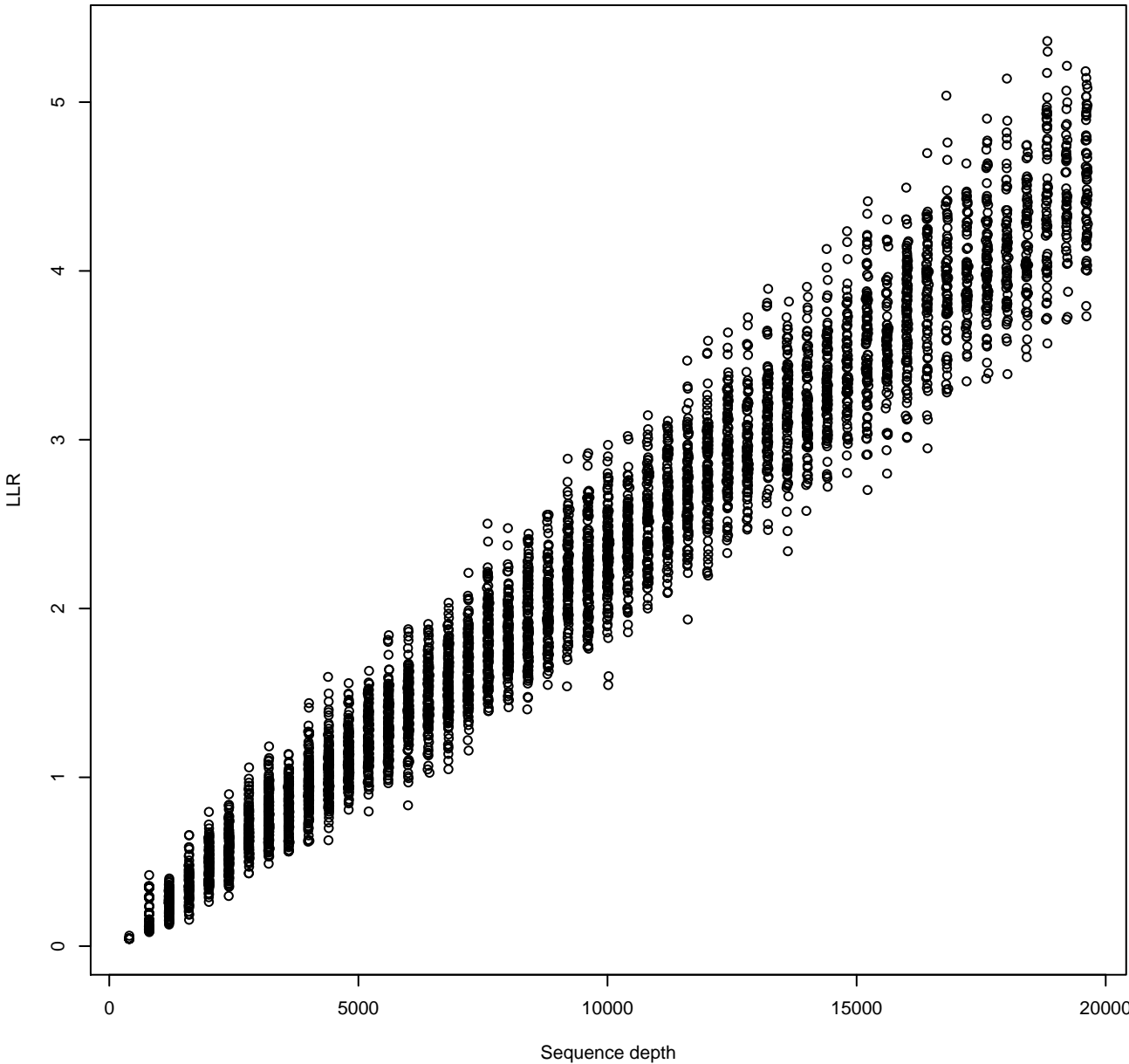

Simulation of site MAF 0.298%~0.302%

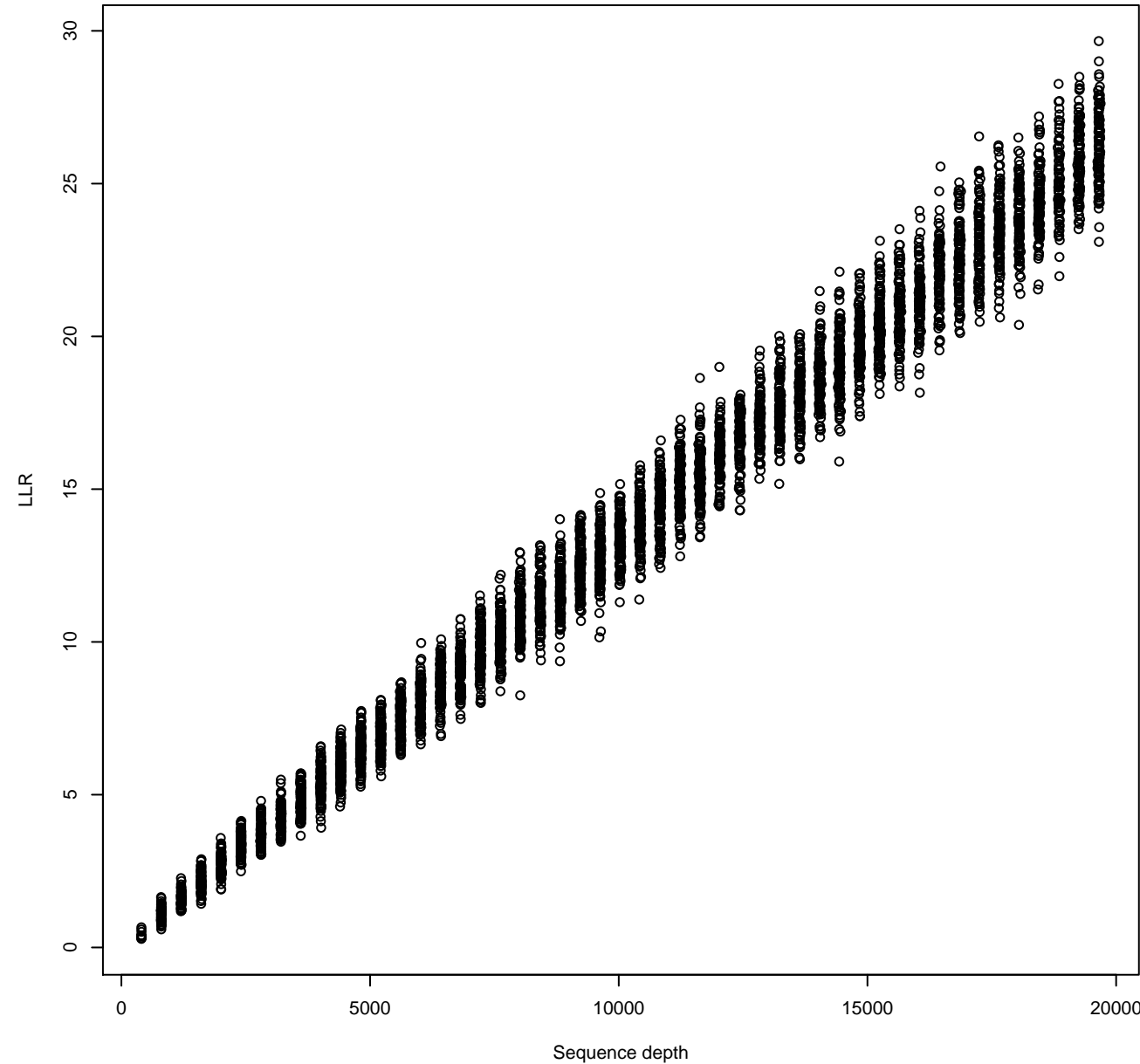

Simulation of site MAF 0.998%~1.002%

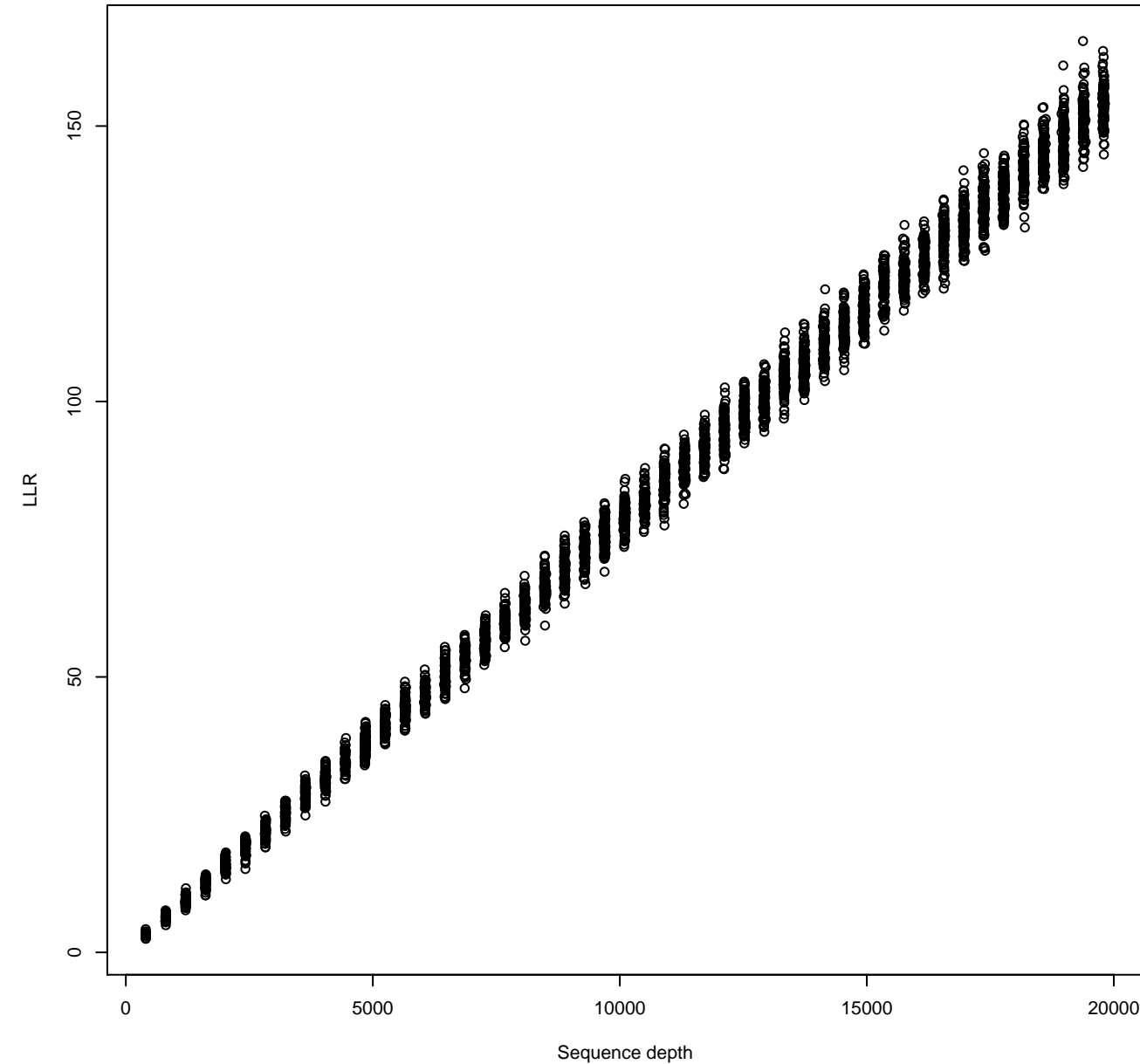

Supplement: Additional file 7: Figure S5. — The sequence depth dependency for minor allele detection in SEGREG-based LLR. Minor alleles with different average levels of MAF (0.1 %, 0.3 % and 1 %) were tested in simulations in each plot. LLR > 3 (from Additional file Figure S4) is used to distinguish true minor alleles from sequence error. (PDF 97 kb) [file 12864_2016_2463_MOESM7_ESM.pdf]

**Segmented Regression, FWSE=0.25**

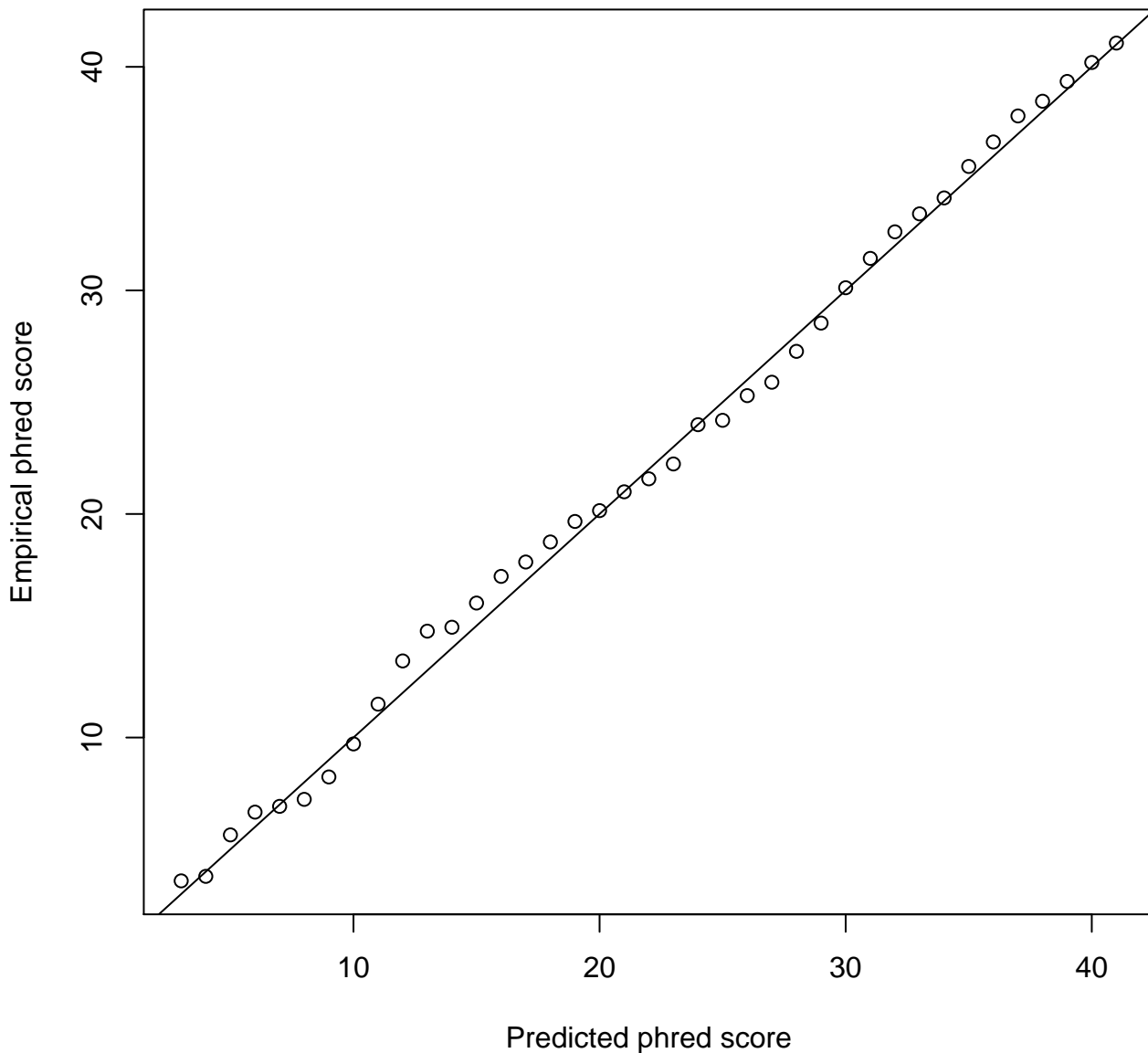

**Quadratic Regression, FWSE=1.11**

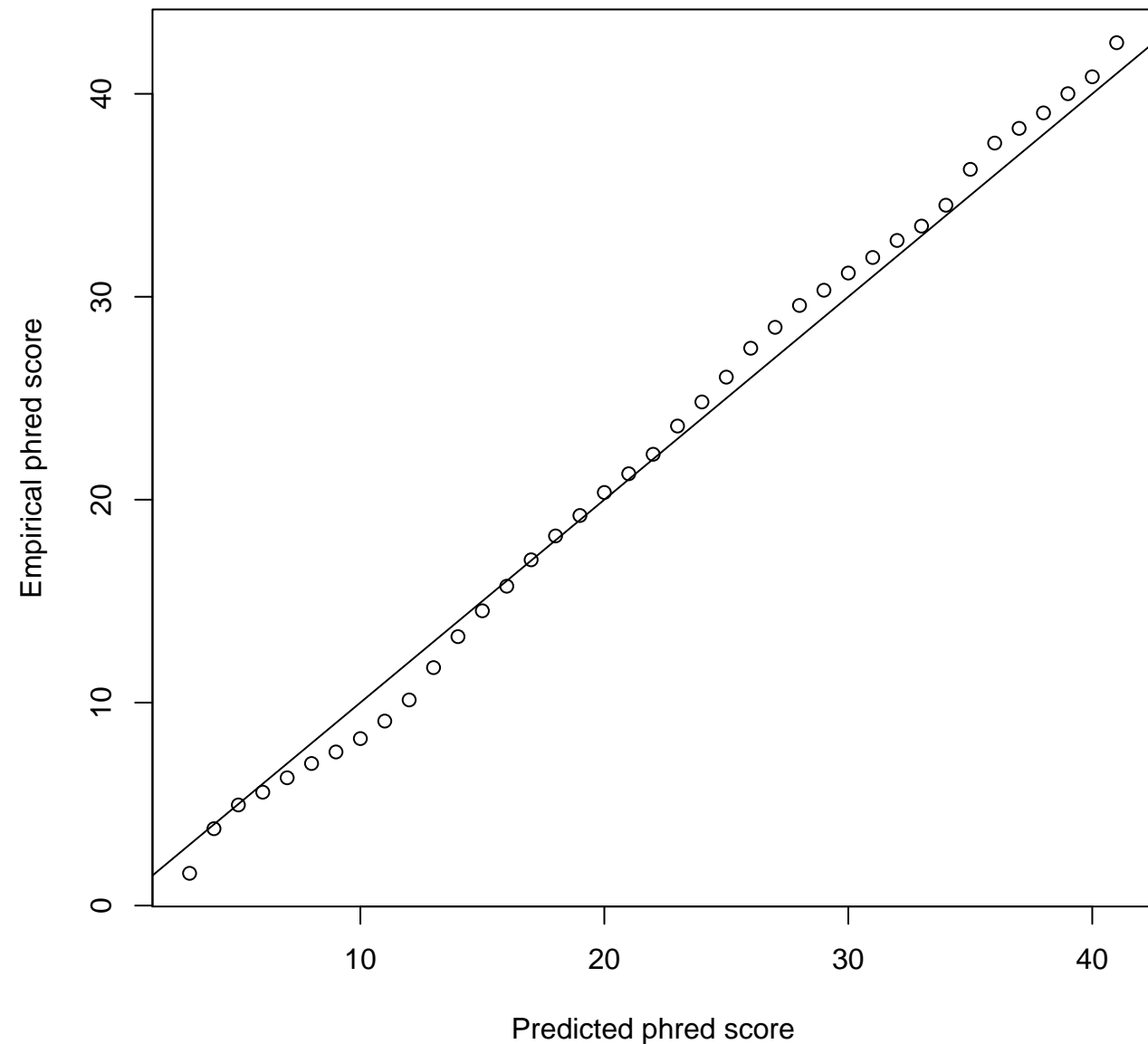

Supplement: Additional file 8: Figure S10. — A comparison of segmented vs. quadratic regression applied to base score recalibration for phiX174 data. Quadratic regression does not result in an overall improvement. (PDF 5 kb) [file 12864_2016_2463_MOESM8_ESM.pdf]

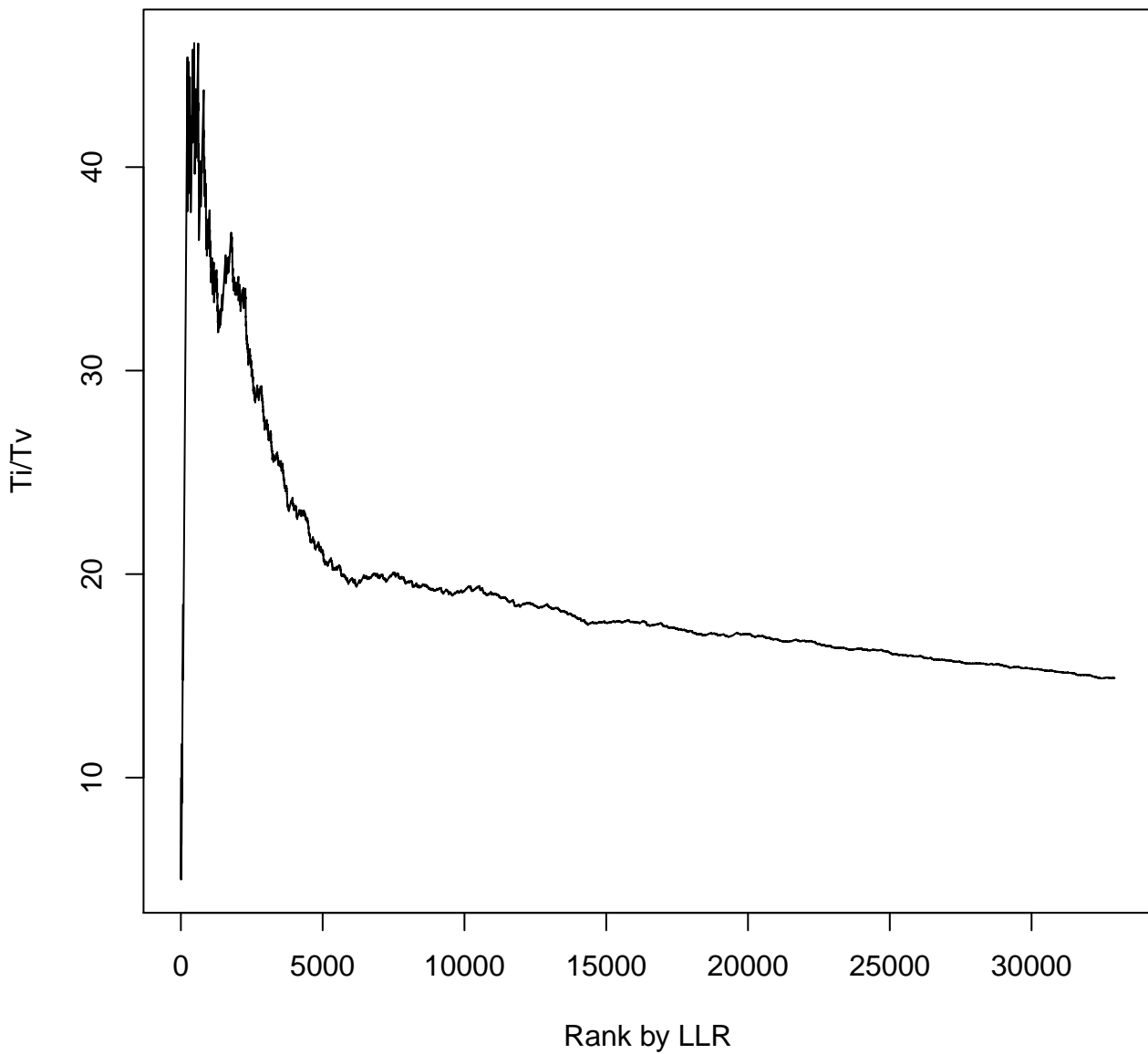

Supplement: Additional file 10: Figure S7. — The cumulative Ti/Tv ratio for minor alleles in mtDNA, ordered according to decreasing LLR. The first 3,000 minor alleles have a Ti/Tv >30, then the ratio decreases to and stabilizes around 18 for the next 5,000 to 30,000 minor alleles. The high Ti/Tv ratio suggests these minor alleles are probably true but have different sources: those with the highest Ti/Tv ratio are from heteroplasmy, which reflects recent mutations, while the rest probably stem from cross contamination or nuMTs, which diverged from the major allele thousands to millions years ago and hence would have a relatively lower Ti/Tv ratio. (PDF 125 kb) [file 12864_2016_2463_MOESM10_ESM.pdf]

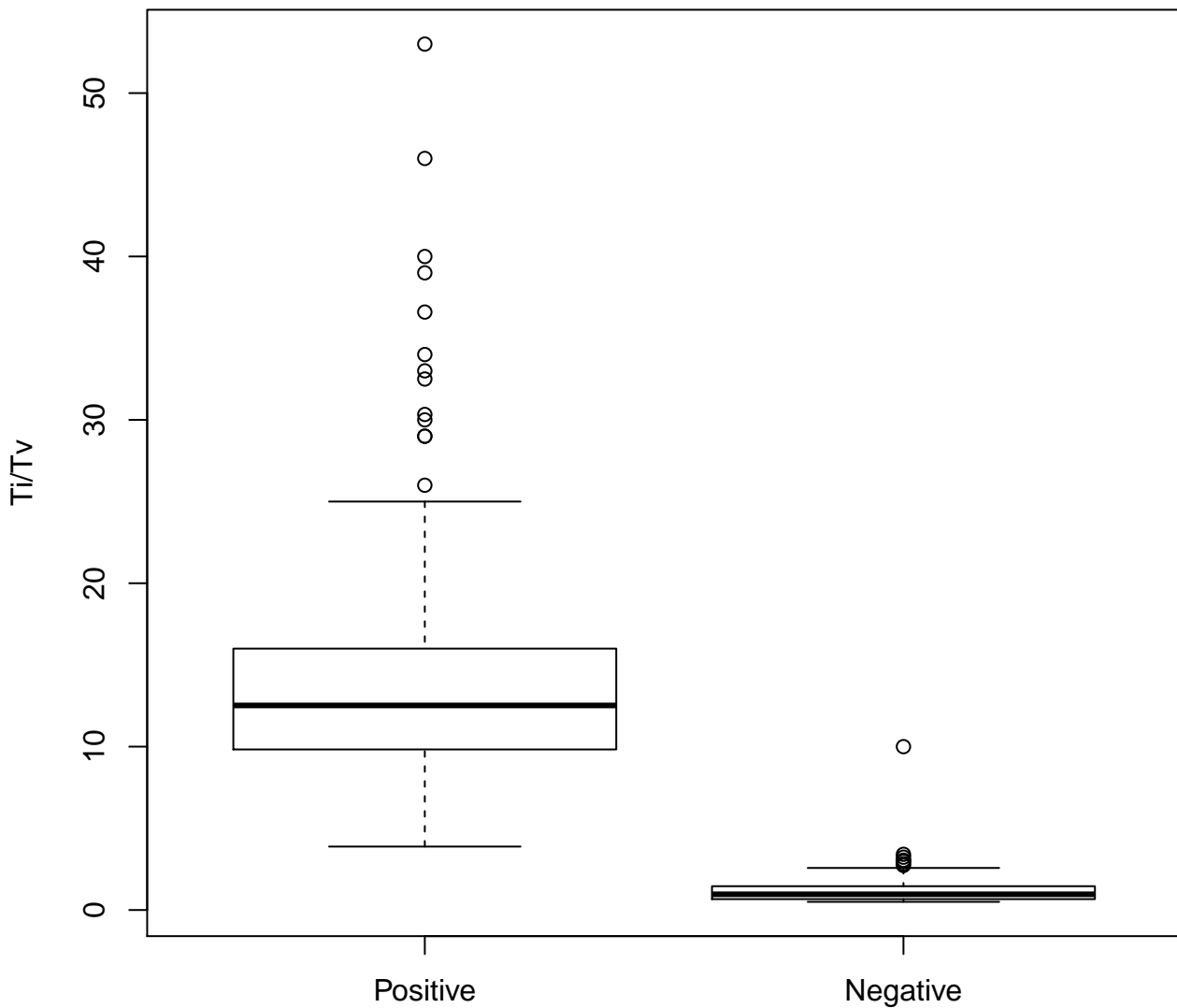

Supplement: Additional file 11: Figure S8. — Ti/Tv ratio among 247 mtDNA samples for minor alleles covered by at least two reads from each strand. Positive set: minor alleles with Segreg based LLR > 3, MAF > 0.001, SB < 1, PosRankSum > -3. Negative set, all other minor alleles. (PDF 4 kb) [file 12864_2016_2463_MOESM11_ESM.pdf]
