# Supplementary material for: Improvement in detection of minor alleles in next generation sequencing by base quality recalibration
Source: BMC Genomics. 2016 Feb 27;17:139. doi: 10.1186/s12864-016-2463-2 (PMC4769523; doi:10.1186/s12864-016-2463-2)
Supplement: Additional file 9: Table S1. — Fifteen minor alleles reported from analysis of phiX174 by various sequence callers. For each analysis, the minor allele is reported only if LLR > 3, otherwise the entry is FAIL. We also omit minor alleles with MAF < 0.1 % in all analyses. The results of the Strand bias test (SB <1) and Position rank sum test (Pos_rank_sum > -3) are also reported. The sequence coverage for each analysis is in the last row of the table. Further notes about the nature of the minor allele are provided in the last column of the table. (PDF 45 kb) [file 12864_2016_2463_MOESM9_ESM.pdf]

Pos	Ref	Consensus	Alt	Strandbias	Pos_rank_	Bustard1		Freeibis1		Freeibis2		Freeibis3		Ibis4		Miseq(Bustard)		notes
						LLR	MAF	LLR	MAF	LLR	MAF	LLR	MAF	LLR	MAF	LLR	MAF	
1401	A	G	A	PASS	PASS	PASS	25.01%	PASS	25.03%	PASS	24.89%	PASS	25.05%	PASS	23.93%	PASS	25.48%	Back mutation or not fixed yet
1644	G	G	A	PASS	PASS	PASS	24.86%	PASS	24.76%	PASS	25.16%	PASS	25.09%	PASS	24.54%	PASS	25.25%	CpG
3035	T	T	G	Filter	Filter	PASS	0.94%	FAIL		FAIL		FAIL		FAIL		FAIL		Filter
3012	G	G	A	Filter	Filter	PASS	0.34%	PASS	0.47%	PASS	0.47%	PASS	0.60%	FAIL		FAIL		Filter
878	C	C	T	PASS	PASS	PASS	0.39%	PASS	0.37%	PASS	0.44%	PASS	0.42%	PASS	0.41%	PASS	0.45%	CpG
3021	G	G	C	Filter	Filter	FAIL		PASS	0.33%	PASS	0.34%	PASS	0.41%	FAIL		FAIL		Filter
670	C	C	A	Filter	Filter	FAIL		FAIL		FAIL		FAIL		PASS	0.26%	FAIL		Filter
912	A	A	C	PASS	PASS	PASS	0.20%	PASS	0.19%	PASS	0.18%	PASS	0.18%	PASS	0.16%	PASS	0.14%	AC sequence error hotspot
3018	A	A	C	Filter	Filter	FAIL		PASS	0.18%	PASS	0.09%	PASS	0.11%	FAIL		FAIL		Filter
587	G	A	C	PASS	PASS	PASS	0.17%	PASS	0.16%	PASS	0.16%	PASS	0.17%	PASS	0.10%	PASS	0.10%	AC sequence error hotspot
5349	C	C	T	PASS	PASS	PASS	0.16%	PASS	0.15%	PASS	0.11%	PASS	0.14%	PASS	0.05%	PASS	0.12%	CpG
4069	G	G	T	Filter	Filter	FAIL		FAIL		FAIL		FAIL		PASS	0.13%	FAIL		Filter
3023	T	T	G	Filter	Filter	PASS	0.13%	FAIL		FAIL		FAIL		FAIL		FAIL		Filter
2339	C	C	T	PASS	PASS	PASS	0.10%	PASS	0.10%	PASS	0.14%	PASS	0.13%	PASS	0.04%	PASS	0.14%	Undetermined
3378	A	A	C	PASS	PASS	PASS	0.10%	PASS	0.09%	PASS	0.09%	PASS	0.11%	PASS	0.09%	PASS	0.11%	AC sequence error hotspot
median sequence coverage						85784		85974		124928		119929		76978		17930		

MAF is estimated by maximum logarithm likelihood

Bustard1 and Freeibis1 are from the same run with different base caller

Freeibis1 and Freeibis2 are from the two lanes of the same flowcell

Iibs is conbined from 8 lanes of the same flowcell

Miseq is conbined from from two independent runs for better sequence coverage
